# A Novel Zeolite–Carbon Nanotube Composite Electrode for the Electrochemical Analysis of Agomelatine in Real Samples

**DOI:** 10.3390/nano15231781

**Published:** 2025-11-26

**Authors:** Katarzyna Fendrych, Wiktoria Głowacz, Joanna Smajdor-Baran, Bogusław Baś

**Affiliations:** Department of Analytical Chemistry and Biochemistry, Faculty of Materials Science and Ceramics, AGH University of Krakow, Al. A. Mickiewicza 30, 30-059 Kraków, Polandsmajdorj@agh.edu.pl (J.S.-B.); bas@agh.edu.pl (B.B.)

**Keywords:** agomelatine, multi-walled carbon nanotubes, voltammetry, zeolite Y, zeolite-modified electrode

## Abstract

This study aimed to develop and apply a novel zeolite-modified electrode (ZME), integrating Cu-exchanged zeolite Y (Cu-ZY) with a conductive carbon matrix composed of multi-walled carbon nanotubes (MWCNTs), for the sensitive and selective voltammetric determination of agomelatine (AGO), an important antidepressant, the accurate determination of which in pharmaceutical and biological samples is critical for therapeutic monitoring and quality control. Drop-casting the Cu-ZY/MWCNTs composite onto the surface of a glassy carbon electrode (GCE) resulted in the formation of a unique sensing platform, which exhibited a significantly improved electrochemical response for the oxidation of AGO. The enhanced activity of Cu-ZY/MWCNTs-GCE, attributed to the synergistic combination of Cu-ZY and MWCNTs, was confirmed by morphological, textural, and voltammetric analyses. Differential pulse voltammetry (DPV) was utilized for the quantitative determination of AGO, with optimization performed on instrumental parameters, supporting electrolyte pH, and preconcentration time (*t_acc_*). Using the Britton–Robinson buffer (BRB) solution at pH 3.0, the Cu-ZY/MWCNTs-GCE exhibited a linear response to AGO concentrations ranging from 8.2 × 10^−9^–9.6 × 10^−7^ mol L^−1^ (0.002–0.23 mg L^−1^), achieving a detection limit (LOD) of 4.3 × 10^−9^ mol L^−1^ (1.04 µg L^−1^) with a preconcentration time of 60 s. The successful determination of AGO in pharmaceutical formulations, wastewater, and biological fluids, with recoveries ranging from 98.0 to 113.0%, demonstrates the effectiveness and practical applicability of the Cu-ZY/MWCNT-GCE-based voltammetric method for agomelatine analysis in complex matrices.

## 1. Introduction

Agomelatine (AGO), chemically known as N-[2-(7-methoxy-1-naphthalenyl)ethyl]acetamide, is a new antidepressant with a unique pharmacological profile, acting as a melatonin receptor agonist (MT1 and MT2) and a selective receptor antagonist for serotonin 5-HT2C [1]. It was first approved for clinical use by the Committee for Medicinal Products for Human Use of the European Medicines Agency in February 2009 for the treatment of major depressive disorder (MDD) in adults [2]. The approval was based on clinical studies that demonstrate its efficacy in alleviating depressive symptoms, regulating circadian rhythms, and improving mood and sleep quality, with a relatively favorable safety profile compared to conventional antidepressants [3].

Due to its increasing clinical importance, the accurate determination of agomelatine in pharmaceutical formulations, biological fluids, and environmental samples has become crucial for quality control, pharmacokinetic studies, and the monitoring of therapeutic drugs. In this context, several analytical techniques have been employed for the quantification of AGO. Chromatographic methods, such as high-performance liquid chromatography (HPLC) and liquid chromatography–mass spectrometry (LC-MS), provide high sensitivity and selectivity and are widely used in pharmacokinetic investigations [4,5,6]. Spectrophotometric [7,8,9] and spectrofluorimetric [10,11] methods offer simpler and cost-effective alternatives, although they generally exhibit lower sensitivity. In recent years, electrochemical methods, particularly voltammetric techniques, have emerged as promising, rapid, and economical alternatives for AGO analysis. The electroactive nature of AGO, attributed to its electron-rich functional groups, such as the naphthalene moiety and the acetamide side chain, allows for detection at low concentrations with high precision. Furthermore, the sensitivity and selectivity of voltammetric assays can be significantly enhanced using chemically modified electrodes, which facilitate electron transfer and improve overall analytical performance. Several voltammetric sensors have been developed for the electrochemical determination of AGO exploring different types of advanced materials, such as CeO_2_ nanoparticles and g-C_3_N_4_ sheets [12], multiwalled carbon nanotubes/cellulose/tween composite [13], cobalt nanoparticles and sugar polymer [14], FeZn-layered double hydroxide/graphene/polyaniline nanocomposite [15], and copper nanoparticles incorporated into a covalent organic framework [16]. Importantly, zeolites have never been utilized in the voltammetric detection of AGO, which highlights their novelty and strategic value in sensor development. The unique crystalline, microporous structure of zeolites, combined with a high surface area and excellent adsorption capabilities, offers the opportunity to improve the preconcentration of the analytes at the electrode interface [17,18]. This not only increases the sensitivity and selectivity of the sensor but also contributes to greater stability and reproducibility of the measurements. Furthermore, the tunable pore size, ion-exchange properties, and chemical stability of the zeolite structure enable effective interaction with AGO molecules while minimizing interference from coexisting compounds. Integrating zeolites with carbon-based conductive nanomaterials, such as graphene oxide [19,20,21], carbon black [22,23,24,25], mesoporous carbon [26,27], and carbon nanohorns [28], can further improve electron transfer kinetics, facilitate faster response times, and provide a versatile platform for designing highly efficient and reliable voltammetric sensors.

Building upon these insights, a novel voltammetric sensor was developed by combining Cu-exchanged zeolite Y (Cu-ZY) with multi-walled carbon nanotubes (MWCNTs) in a modifying layer deposited onto the surface of a glassy carbon electrode (GCE) via the drop-casting method. The resulting Cu-ZY/MWCNTs-GCE sensor was subsequently applied for the electrochemical determination of agomelatine in different samples and in the presence of numerous interfering species. The synergistic characteristics of the Cu-ZY/MWCNTs nanocomposite, resulting from the combination of catalytic, conductive, and electrochemically stable components, were verified through structural and surface analyses using X-ray fluorescence (XRF), nitrogen adsorption–desorption isotherms, and scanning electron microscopy/energy-dispersive X-ray spectroscopy (SEM/EDS) techniques. To develop a sensitive, selective, and robust approach for AGO determination, the experimental conditions of differential pulse voltammetry (DPV), including instrumental parameters and the pH of the supporting electrolyte, were systematically optimized. Comprehensive analytical evaluation, including linear range, detection limit, and both short- and long-term stability, demonstrated performance comparable to or exceeding previously reported methods. Finally, the optimized protocol was successfully applied for the quantitative determination of AGO in pharmaceutical formulations, environmental water samples, and biological fluids, illustrating its versatility and practical utility across diverse matrices.

## 2. Experimental Section

### 2.1. Chemicals and Solutions

Zeolite Y in the sodium form (CBV 100) was purchased from Zeolyst International (Conshohocken, PA, USA). Multi-walled carbon nanotubes (MWCNTs, >90% carbon purity, D × L = 110–170 nm × 5–9 μm) were received from Sigma-Aldrich (St. Louis, MO, USA) and used as received without further treatment. Dichloromethane (ACS reagent), acetone (HPLC grade, ≥99.9%), and polystyrene (Mw ≈ 35,000) were obtained from Honeywell Research Chemicals (Seelze, Germany), Avantor Performance Materials Poland S.A. (Gliwice, Poland), and Sigma-Aldrich, respectively. Copper(II) nitrate trihydrate (puris. p.a., 99–104%) was purchased from Sigma-Aldrich. Agomelatine (≥98%, HPLC grade, Sigma-Aldrich) was used to prepare a 1000 mg L^−1^ stock solution in double-distilled water on a weekly basis. The solution was stored at 4 °C, protected from light, and diluted as required prior to analysis. Britton-Robinson buffer (BRB) solutions with pH values ranging from 2.0 to 4.0 were prepared by mixing equimolar amounts (0.04 mol L^−1^ each) of acetic acid, boric acid, and phosphoric acid (Avantor Performance Materials Poland S.A.), followed by adjustment of the pH with an appropriate volume of 0.2 mol L^−1^ NaOH solution (Avantor Performance Materials Poland S.A.). Titanium dioxide, starch, cellulose, and magnesium stearate (Sigma-Aldrich) in the powder form, potassium chloride, sodium phosphate, sodium nitrate, magnesium sulfate heptahydrate, and calcium chloride hexahydrate (Avantor Performance Materials Poland S.A.) at the concentration of 0.1 mol L^−1^, as well as humic acid, uric acid, cetrimonium bromide (CTAB), sodium dodecyl sulfate (SDS), and Triton X-100 (Sigma-Aldrich) at the concentration of 0.5 g L^−1^ were used in interference studies. All reagents were of analytical grade and applied without additional purification. Certified reference materials (CRMs) of wastewater (SPS-WW1, Batch 114 and SPS-WW2, Batch 113) were obtained from Spectrapure Standards AS (Oslo, Norway). Synthetic human plasma and urine were sourced from Biowest (Nuaillé, France) and Medichem Diagnostica GmbH & Co. (Steinenbronn, Germany), respectively.

### 2.2. Apparatus

The chemical composition of zeolite Y was determined by X-ray fluorescence (XRF) using a wavelength-dispersive spectrometer (WD-XRF, Axios mAX 4 kW, PANalytical, Malvern, UK) equipped with a rhodium-target X-ray tube. Semiquantitative analysis of the acquired XRF spectrum was performed using the Omnian standardless evaluation software (PANalytical). Morphological analysis of zeolite Y, MWCNTs, and the modifying layer was performed using a Thermo Scientific Scios 2 DualBeam ultra-high-resolution focused ion beam scanning electron microscopy (FIB-SEM). The elemental composition of zeolite Y, both before and after functionalization with copper ions, was determined using energy-dispersive X-ray spectroscopy (EDS). The nitrogen adsorption–desorption measurements at 77 K (ASAP 2010, Micromeritics, Norcross, GA, USA) were used to evaluate the textural properties of zeolite Y and MWCNTs. Prior to analysis, the samples were degassed at 623 K for 24 h. The Brunauer–Emmett–Teller (BET) and Barrett–Joyner–Halenda (BJH) methods were employed to establish the specific surface areas (SSA) and porosity of modifiers, respectively.

Voltammetric measurements were carried out using a M161 multipurpose electrochemical analyzer equipped with an M164D electrode stand (mtm-anko, Kraków, Poland). Data were recorded in real time using EALab 2.1 software. A 10 mL three-electrode cell comprised a bare or modified glassy carbon electrode (GCE, MF-2012, 3 mm diameter, BASi, Bioanalytical Systems, Inc., West Lafayette, IN, USA) as the working electrode, a double-junction Ag |AgCl| 3 M KCl reference electrode (MINERAL, Gliwice, Poland), and a platinum wire auxiliary electrode. To ensure solution homogeneity, the solutions were magnetically stirred at ~200 rpm using a Teflon^®^-coated stir bar (Kartell, Noviglio, Italy). A SevenCompact S210 pH meter (Mettler Toledo, Greifensee, Switzerland) was used to adjust the pH of the BRB solutions.

### 2.3. Procedures

#### 2.3.1. Functionalization of Zeolite Y with Copper Ions

The functionalization of zeolite involves the partial or complete replacement of the natural cations present in the zeolite Y framework with copper ions via an ion-exchange procedure. In summary, 0.5 g of the sodium form of zeolite Y (Na-ZY) was dispersed in 5 mL of an aqueous solution of copper(II) nitrate (Cu(NO_3_)_2_·3H_2_O) at a concentration of 0.2 mol L^−1^. The mixture was shaken (2500 rpm) at 25 °C for 24 h to allow exchange of native Na^+^ ions with Cu^2+^ ions. After this first treatment, the suspension was centrifuged at 13 500 rpm for 10 min, the supernatant was removed, and the zeolite was treated again with a fresh portion of the copper(II) solution under the same conditions. After the two-step ion exchange, the Cu-form of zeolite Y (Cu-ZY) was washed several times with double-distilled water to remove any residual copper salts and dried at room temperature for 24 h. The successful incorporation of Cu^2+^ ions into the zeolite framework was verified by SEM/EDS analysis.

#### 2.3.2. Preparation of Cu-ZY/MWCNTs Suspension and Electrode Surface Modification

The modifying suspension was prepared using Cu-ZY and multi-walled carbon nanotubes (MWCNTs) connected in a weight ratio of 1:1. Initially, 50 mg of Cu-ZY and 50 mg of MWCNTs were thoroughly ground together in an agate mortar for 10 min to obtain a homogeneous mixture. Subsequently, 10 mg of the Cu-ZY/MWCNTs composite was placed in an Eppendorf tube and dispersed in 500 µL of an acetone: dichloromethane (2:3 *v*/*v*) solvent mixture containing 5 mg of polystyrene as a binder. The suspension was homogenized for 30 min (1800 rpm) using Vortex Multi Speed MSV-3500 (BioSan, Riga, Latvia) to ensure uniform dispersion of the composite.

For electrode modification, a simple drop-casting method was employed. Prior to modification, the surface of the GCE was polished on a polishing pad using 0.3 µm alumina powder (Buehler Micropolish II, Lake Bluff, IL, USA), thoroughly washed with water, and rinsed several times with acetone to achieve a smooth and contaminant-free surface. After that, 10 µL of the prepared Cu-ZY/MWCNTs suspension was carefully deposited onto the GCE surface and allowed to dry under ambient laboratory conditions for 24 h. To evaluate the contributions of the individual components of the composite to the overall electrochemical response, GCEs modified with 10 mg of Cu-exchanged zeolite (Cu-ZY-GCE) or 10 mg of MWCNTs (MWCNTs-GCE) were prepared using the same procedure.

#### 2.3.3. Real Samples Preparation and Analysis

*Agomelatine Adamed* (25 mg per tablet, Adamed Pharma S.A., Czosnów, Poland) was purchased from the local pharmacy. The powder obtained from five finely ground tablets was accurately weighed and dissolved in 25 mL of double-distilled water to obtain a stock solution of agomelatine with a target concentration of 1000 mg L^−1^. The resulting solution was ultrasonicated for 15 min and then filtered through a 0.22 µm mixed cellulose ester (MCE) syringe filter (Alchem Grupa Sp. z o.o., Nowa Sól, Poland) to remove any insoluble excipients. For voltammetric analysis, the filtered solution was first diluted 100 times with double-distilled water. An aliquot of 20 µL of this diluted sample was then introduced into a voltammetric cell containing 5 mL of Britton Robinson buffer (pH 3.0). Agomelatine concentration was determined using the standard addition approach with four consecutive scans recorded at each step. A background correction was applied, and the average peak current was used to calculate the agomelatine content in the analyzed pharmaceutical, accounting for the dilution factor.

Wastewater and human urine samples in the form of CMR were first filtered through a 0.22 µm MCE syringe filter to remove suspended particles. The filtrates were spiked with agomelatine stock solution (100 mg L^−1^) to achieve a concentration of 1 mg L^−1^ for wastewater and 5 mg L^−1^ for urine. Appropriate aliquots of the spiked samples were added to the supporting electrolyte to obtain a final agomelatine concentration of 50 µg L^−1^ in the voltammetric cell. DPV measurements were performed using the same standard addition protocol, and the accuracy of the method was verified by calculating recovery values. The recovery test was also performed for a synthetic human plasma sample spiked with agomelatine standard solution (100 mg L^−1^) to achieve a concentration of 5 mg L^−1^. Protein components in the spiked plasma, which could affect the voltammetric response, were eliminated through a precipitation step involving the addition of 200 µL of 10% trichloroacetic acid to 800 µL of the plasma sample, followed by vortexing for 5 min and centrifugation at approximately 14,500 rpm for 30 min (Eppendorf, Hamburg, Germany). The resulting supernatant was then filtered through a 0.22 µm syringe filter, appropriately diluted with Britton–Robinson buffer (pH 3.0) to a final concentration of 50 µg L^−1^ in the voltammetric cell, and analyzed using the standard addition method described above.

#### 2.3.4. Standard Procedure of Voltammetric Measurements

Differential pulse voltammetry (DPV) was employed for the quantitative measurements of agomelatine concentration on the Cu-ZY/MWCNTs-GCE. Measurements were carried out in 0.04 mol L^−1^ Britton–Robinson buffer (BRB) solution at pH 3.0 (total volume of 5 mL) with potential scanned from +0.7 to +1.25 V in both anodic and cathodic directions. with the following optimized parameters: *E_s_* = 5 mV, pulse amplitude *dE* = 40 mV, pulse time *t_imp_* = 20 ms (current sampling time *t_s_* = 10 ms and waiting time *t_w_* = 10 ms). For each measurement, at least four consecutive voltammograms were recorded, and the average peak current was used for quantitative analysis. The removal of dissolved oxygen was unnecessary, as the applied potentials were exclusively positive, under which oxygen remains electrochemically inactive.

## 3. Results and Discussion

### 3.1. Examination of Electrode Characteristics

The zeolite Y is a type of synthetic molecular sieve belonging to the faujasite (FAU) family of zeolites. The pore structure of ZY features supercages approximately 12 Å in diameter, connected by windows around 8 Å in diameter, formed by rings of 12 interconnected tetrahedra (12-rings) [29]. The chemical composition of the analyzed zeolite Y, determined by XRF measurements, was as follows (in wt. %): SiO_2_—62.25, Al_2_O_3_—21.34, Na_2_O—16.05, K_2_O—0.03, MgO—0.09, CaO—0.14, TiO_2_—0.01, Fe_2_O_3_—0.02, and other—0.07. The calculated silica-to-aluminum (Si/Al) molar ratio of 4.9 indicates that ZY is a low-silica zeolite with a high aluminum content. This composition results in a high cation-exchange capacity and a hydrophilic surface character, which promotes the adsorption of polar, electroactive molecules on the electrode surface.

The textural properties of ZY and MWCNTs (Table 1) significantly influence their suitability for electrode modification. Zeolite Y exhibits an exceptionally high BET surface area, providing abundant active sites for electroactive species and potentially enhancing their accumulation and selectivity in modified electrodes. In contrast, MWCNTs present a much higher external surface area and wider pore widths, offering efficient electron transfer, which makes them excellent candidates for enhancing electrode conductivity and facilitating fast electrochemical kinetics.

**Figure 1:**
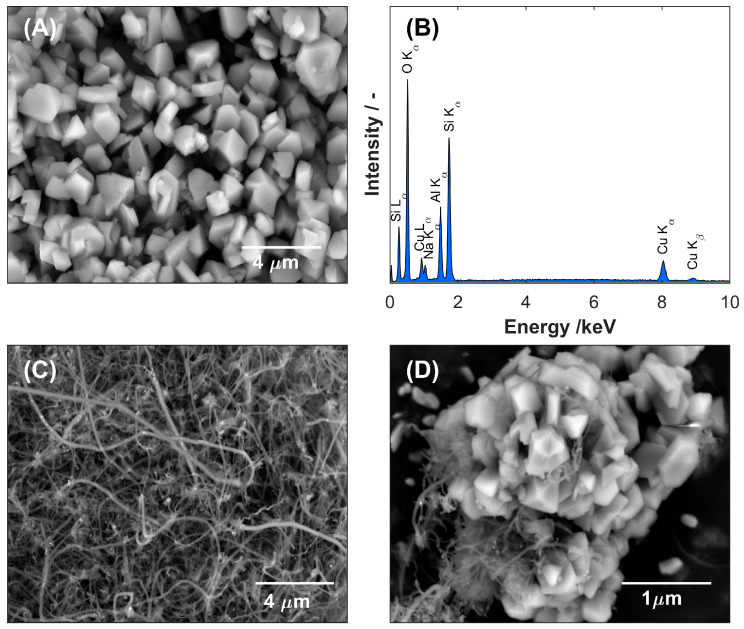
SEM image of (**A**) Cu-zeolite Y with (**B**) the corresponding EDS spectrum, (**C**) MWCNTs, and (**D**) Cu-ZY/MWCNTs modifying layer deposited on the surface of GCE.

The SEM image of zeolite Y (Figure 1A) shows densely packed, well-defined crystalline particles with predominantly faceted morphologies. The crystallites show the characteristic polyhedral geometry of faujasite-type zeolites and appear to have a relatively uniform size distribution within the sub-micrometer range. Individual particles form a compact and continuous modification layer that fully covers the GCE substrate and confirms successful immobilization of the zeolite coating. At the same time, the EDS analysis of zeolite Y performed before and after the ion-exchange process confirmed the effective substitution of sodium cations with copper ions. This replacement was evidenced by the marked reduction in the intensity of the Na peak with the simultaneous appearance of characteristic Cu peaks (Lα and Cu Kα/Kβ) in the EDS spectrum of Cu-ZY (Figure 1B). The copper content in the ZY structure was quantified by averaging the atomic concentrations obtained from multiple surface points, resulting in a value of 7.6% at. The SEM observation of the MWCNTs-modified layer (Figure 1C) reveals a dense, interconnected network of entangled nanotubes uniformly coating the surface of GCE. The nanotubes display high aspect ratios, smooth surfaces, and multiple concentric walls, characteristic of multi-walled structures. In addition, minor bundling of nanotubes can be observed, likely as a result of van der Waals interactions, which contributes to the mechanical stability of the modified layer while maintaining its overall high conductivity and electrochemical activity. Finally, in the morphology of the Cu-ZY/MWCNTs modifying layer (Figure 1D) within the entangled network of nanotubes, Cu-ZY zeolite particles are distinctly observed as dispersed granular clusters, adhered to, and partially embedded within the nanotube matrix.

**Figure 2:**
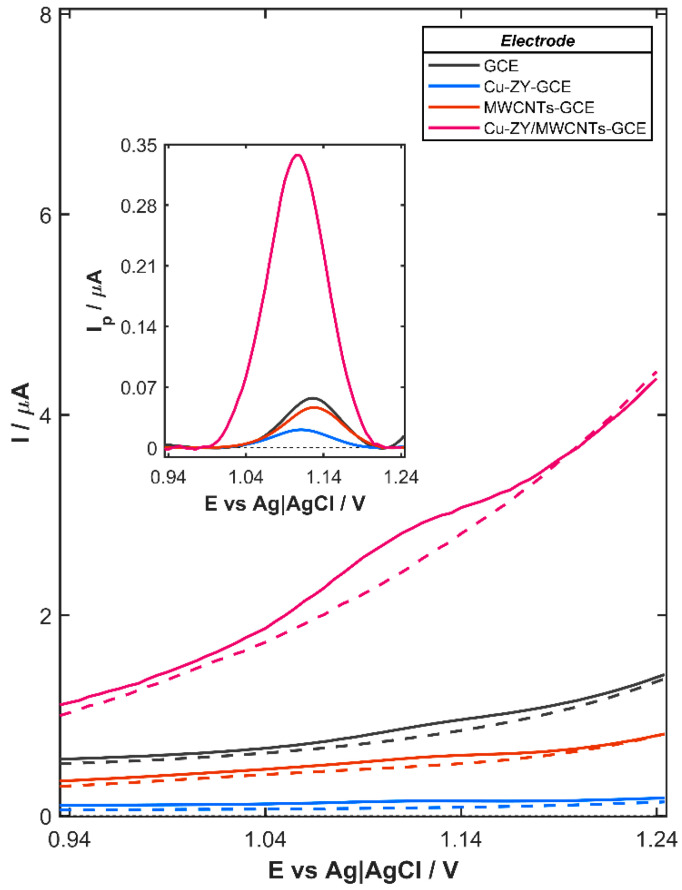
Comparison of DPV curves recorded on the bare GCE, Cu-ZY-GCE, MWCNTs-GCE, and Cu-ZY/MWCNTs-GCE in 0.04 mol L^−1^ BRB solution (pH 3.0) containing 80 µg L^−1^ agomelatine (dashed lines denote the background signal). Inset: DP voltammograms subjected to the background correction. Experimental conditions: *E_s_* = 5 mV, *dE* = 40 mV, *t_imp_* = 20 ms (*t_w_* = *t_s_* = 10 ms), and *t_acc_* = 0 s.

Synergistic integration of the complementary properties of Cu-ZY and MWCNTs results in a significantly improved electrochemical performance of the Cu-ZY/MWCNTs-modified GCE toward agomelatine oxidation, as demonstrated by the DPV response recorded in the presence of 80 µg L^−1^ AGO in 0.04 mol L^−1^ BRB solution (pH 3.0). As shown in Figure 2, the bare GCE, which serves as the substrate for the modifying layer, exhibited a discernible AGO oxidation signal, which becomes more pronounced after background subtraction (inset in Figure 2). A similar behavior is observed for GCE modified with the layer containing only Cu-ZY (Cu-ZY-GCE) or MWCNTs (MWCNTs-GCE), indicating that neither component alone is sufficient to significantly enhance the electrochemical signal. The relatively low response observed for the Cu-ZY-GCE (0.021 µA) is attributed to the intrinsic properties of the zeolites. Although Cu-ZY provides catalytic sites, its overall electrical conductivity is limited due to the insulating nature of the aluminosilicate framework. As a result, electron transfer between the electrode surface and agomelatine molecules is inefficient, leading to a weak oxidation signal. On the contrary, while MWCNTs offer high electrical conductivity, the electrochemical response of MWCNTs-GCE also remains relatively low and is equal to 0.046 µA. This behavior can be associated with the absence of specific catalytic or adsorption sites for the analyte, which limits interaction between the AGO molecules and the electrode surface, thereby diminishing the efficiency of the oxidation process. The combination of Cu-ZY and MWCNTs overcomes these individual limitations, producing a synergistic effect that significantly improves electron transfer and interaction with AGO. Consequently, the oxidation signal on Cu-ZY/MWCNTs-GCE (0.338 µA) is greatly enhanced, reaching values nearly 12- and 6-times higher than those recorded on Cu-ZY-GCE and MWCNTs-GCE, respectively. Furthermore, the catalytic effect of Cu-ZY is clearly demonstrated by the shift in the oxidation peak potential from +1128 mV for the bare GCE to +1105 mV for Cu-ZY/MWCNTs-GCE. This significant reduction in the overpotential indicates facilitated electron transfer due to the lower energy barrier for the oxidation of AGO, which contributes to enhanced sensitivity and selectivity of the sensor.

The enhanced electrochemical response of Cu-ZY/MWCNTs-GCE can be further explained by the specific interactions between AGO molecules and Cu ions presented within the zeolite framework, which act as Lewis acid sites capable of transferring electrons to adsorbed molecules [30]. These Cu sites interact with electron-rich functional groups of agomelatine, promoting its adsorption and increasing the local concentration of AGO near the conductive MWCNTs network, thereby enhancing electron transfer during oxidation. Moreover, Cu species in zeolites can undergo changes in oxidation between Cu^2+^ and Cu^+^ states, enabling them to act as effective catalysts in numerous oxidation reactions [31]. The coordinated Cu centers may also directly participate in the electrocatalytic oxidation of AGO by transiently accepting electrons from the molecule, lowering the overpotential for the oxidation process. Such a dual role of Cu ions, enhanced adsorption via Lewis acid-base interactions and catalytic promotion of electron transfer [32], provides a possible explanation for the observed synergistic enhancement, complementing the high conductivity and electron transport offered by MWCNTs

### 3.2. Optimization of the Experimental Conditions

The high sensitivity, low detection limit, excellent peak resolution, and reproducible measurement of small current changes associated with analyte redox reactions make differential pulse voltammetry (DPV) particularly advantageous for the quantitative determination of agomelatine. To ensure the most suitable experimental conditions for the determination of AGO, the pH of the supporting electrolyte and key instrumental parameters of the DPV technique were systematically optimized using a univariate approach. Peak current measurements for 80 µg L^−1^ AGO were repeated three times at each value of the varying parameter, and the associated standard deviation was evaluated. Based on the shape, symmetry, and height of the recorded signal, the most appropriate value of pH of the BRB solution and step potential (*E_s_*), pulse amplitude (*dE*), pulse time (*t_acc_*), and accumulation time (*t_acc_*) was chosen.

#### 3.2.1. pH Value of Supporting Electrolyte

The influence of the pH of 0.04 mol L^−1^ Britton-Robinson buffer solution on the electrochemical response of agomelatine (80 µg L^−1^), including peak potential and peak current, was investigated over the pH range of 2.0 to 4.0. The results indicate that within this pH range, both the peak current and peak potential of AGO remained essentially unchanged. This behavior is consistent with the very high *pK_a_* reported for agomelatine (~15.96) for the relevant acidic functional group, indicating that the molecule remains fully protonated within this acidic range. As the electroactive species does not undergo changes in protonation between pH 2 and pH 4, no significant variation in either peak potential or peak current is observed. These results suggest that within this pH region, the redox process of agomelatine is independent of proton concentration and proceeds without proton-coupled electron transfer. The electroactive form of agomelatine remains stable and dominant under these conditions, implying that the electrode reaction on the Cu-ZY/MWCNTs-GCE is governed primarily by electron transfer.

Among the media tested, the BRB solution at pH 3.0 provided the most stable and intense voltammetric response. At this pH, AGO remains predominantly protonated, which enhances its electrostatic attraction to the negatively charged surface of the modified electrode and promotes more efficient pre-concentration of the analyte. Moreover, acidic conditions stabilize the redox-active Cu species in the Cu-ZY catalyst and facilitate proton-coupled electron-transfer steps [33] that govern the electrocatalytic oxidation of AGO. At higher pH values, both the protonation state of AGO and the stability of the Cu active sites are less favorable, resulting in decreased oxidation currents. Accordingly, the choice of pH 3.0 as the supporting electrolyte represents an optimal compromise, providing both maximal signal intensity and reproducibility, while maintaining the chemical and electrochemical stability of the analyte and the electrode surface.

#### 3.2.2. Optimization of DPV Instrumental Parameters

**Figure 3:**
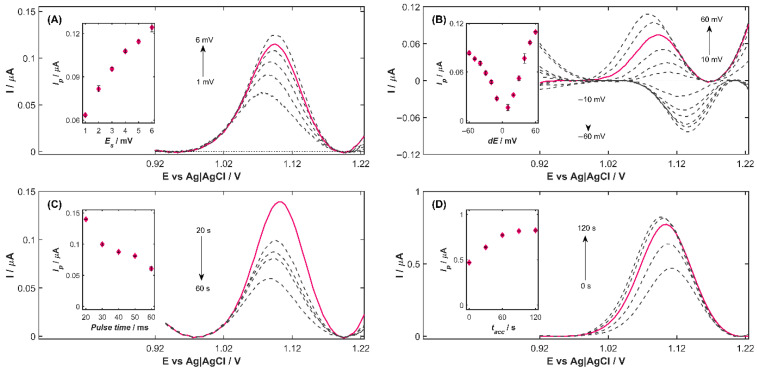
DP voltammograms recorder on Cu-ZY/MWCNTs-GCE in BRB solution (pH 3.0) containing 80 µg L^−1^AGO after baseline correction for different values of DPV parameters: (**A**) step potential *E_s_*, (**B**) pulse amplitude *dE*, (**C**) pulse time (*t_imp_*), and (**D**) accumulation time *t_acc_*. Inset: influence of each optimized parameter on the agomelatine oxidation peak current.

To select the most appropriate potential step (*E_s_*), DPV curves were recorded at step values ranging from 1 to 6 mV (Figure 3A) while maintaining a pulse time of 40 ms and a pulse amplitude of 40 mV under the previously established measurement conditions. The results demonstrate that increasing the step potential value leads to a pronounced enhancement of the peak current. Specifically, raising *E_s_* from 1 to 6 mV produced an increase in peak current from 0.0635 to 0.1242 μA, corresponding to a 95.5% improvement of the agomelatine oxidation signal. The dependence of the peak current *I_p_* (after background subtraction) on the step potential height (inset in Figure 3A) initially exhibits a linear relationship. As *E_s_* increases further, the increments in the peak current become progressively smaller, and the *I_p_* (*E_s_*) function begins to level off. At the same time, a noticeable broadening of the half-peak width is observed at higher *E_s_* values. From these observations, *E_s_* = 5 mV (pink line in Figure 3A) was identified as the optimal step potential for agomelatine determination using the Cu-ZY/MWCNTs-GCE, providing a well-defined, reproducible peak without significant loss of signal intensity, and was applied in subsequent experiments.

For pulse amplitude (*dE*) optimization, *dE* values ranging from 10 to 60 mV in both positive and negative modes were tested using the previously selected *E_s_*. As shown in Figure 3B, the oxidation peak potential of AGO shifted toward more negative potentials with positive pulse amplitudes, whereas negative pulse amplitudes induced a shift in the peak potential toward more positive values. Simultaneously, higher pulse amplitude values lead to less reproducible measurement results. The dependence of the peak current on the pulse amplitude (inset in Figure 3B) indicated that *I_p_* of AGO increased with the absolute value of *dE* in both negative and positive modes. However, at equivalent magnitudes, positive pulse amplitudes consistently produced higher peak currents than negative ones, indicating better sensitivity and signal reproducibility in the positive mode. Taking into account the aforementioned factors, the value of *dE* = +40 mV (pink line in Figure 3B) was considered optimal for the determination of AGO.

Pulse time (*t_imp_*), defined as the sum of waiting time (*t_w_*) and current sampling time (*t_p_*), was selected based on the DPV curves (Figure 3C) recorded for five *t_imp_* values (20, 30, 40, 50, and 60 ms) using the previously selected step potential of 5 mV and a pulse amplitude of 40 mV. The main purpose of using the waiting time is to reduce the background current (*I_b_*), whose primary component is the capacitive current. It can be noted that increasing the pulse time resulted in lower peak current values, as reflected in the decreasing trend of the peak current versus pulse time relationship (inset in Figure 3C). Considering both the peak current height and the repeatability of the measurements, the optimal *t_imp_* was found to be 20 ms, corresponding to 10 ms of waiting time and 10 ms of current sampling time. This value was used in all subsequent measurements.

The accumulation time (*t_acc_*) plays a critical role in the enhancement of the analytical signal in voltammetric measurements by allowing the analyte to pre-concentrate on the electrode surface. To establish the optimal *t_acc_* for agomelatine determination, the current response of Cu-ZY/MWCNTSs-GCE was recorded in the presence of 80 µg L^−1^ AGO over an accumulation time range of 0–120 s, using the previously optimized parameters: *E_s_* = 5 mV, *dE* = 40 mV, *t_w_* = 10 ms, and *t_s_* = 10 ms (Figure 3D). As indicated by the results, increasing *t_acc_* from 0 to 60 s led to a proportional increase in the peak current (inset in Figure 3D), reflecting enhanced AGO accumulation and more efficient electron transfer at the surface of Cu-ZY/MWCNTs-GCE. For *t_acc_* = 60 s, the analytical signal was 64% higher compared to measurements performed without the preconcentration step (*t_acc_* = 0 s). Extending the accumulation time beyond 60 s results in a negligible signal enhancement, with the AGO peak height reaching a gradual stabilization, suggesting that the electrode surface becomes nearly saturated with AGO. Therefore, *t_acc_* = 60 s was chosen as the optimal accumulation time, since it ensures a considerable increase in peak current while maintaining a reasonable analysis duration. This condition provides the best balance between sensitivity improvement and practical efficiency for the voltammetric determination of AGO.

### 3.3. Analytical Performance

**Figure 4:**
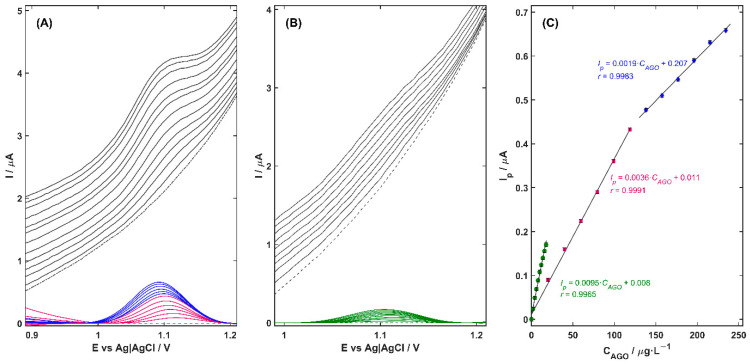
DPV response of Cu-ZY/MWCNTs-GCE registered in 0.04 mol L^−1^ BRB solution (pH 3.0) for the increasing concentration of AGO in the range from blank (dashed line) to (**A**) 0.23 mg L^−1^, and (**B**) 0.18 mg L^−1^ (black lines) with background-corrected curves (colored lines). (**C**) Corresponding calibration plots. Experimental condition: *E_s_* = 5 mV, *dE* = 40 mV, *t_imp_* = 20 ms (*t_w_* = *t_s_* = 10 ms), *t_acc_* = 0 s (**A**) and *t_acc_* = 60 s (**C**).

Under optimized conditions, the analytical performance of Cu-ZY/MWCNTs-GCE in 0.04 mol L^−1^ BRB solution (pH 3.0) for the quantification of AGO was investigated over a series of increasing analyte concentrations using the DPV technique, under two modes of operation: direct detection and detection following preconcentration on the electrode surface (Figure 4A,B, respectively). Background-corrected voltammograms, shown as colored curves in Figure 4, were obtained by subtracting the signal recorded in the blank electrolyte (dashed line). From these, linear calibration plots of peak current versus AGO concentration were constructed (Figure 4C), and the resulting analytical parameters, including sensitivity, linear range (*LR*), limit of detection (*LOD*), and limit of quantification (*LOQ*), are summarized in Table 2. The *LOD* and *LOQ* values were calculated using the standard formulas: *LOD* = 3.3·*SD*/*b* and *LOQ* = 10·*SD*/*b*, where *SD* is the standard deviation of the *y*-intercepts of the regression line, and *b* is the slope of the calibration curve.

As can be noted, the Cu-ZY/MWCNTs-GCE exhibits a pronounced linear correlation between the oxidation peak current and AGO concentration throughout all tested concentration ranges. In the absence of a preconcentration step (*t_acc_* = 0 s, Figure 4A), the AGO peak current increased linearly over two concentration ranges—the first from 20.0 to 118.6 µg L^−1^ (pink curves) and the second from 138.1 to 234 µg L^−1^ (blue curves) with a correlation coefficient (*r*) of 0.9991 and 0.9983, respectively. Extending the preconcentration time to 60 s (Figure 4B) had a significant impact on the analytical performance of the Cu-ZY/MWCNTs-GCE sensor. A nearly threefold increase in sensor sensitivity was obtained, as reflected by the calibration slope that increased from 0.0036 to 0.0095 µA L µg^−1^. This improvement was accompanied by a significant reduction in both the detection and quantification limits, which reached the value of 4.3·10^−9^ mol L^−1^ and 1.3·10^−8^ mol L^−1^, respectively (Table 2). These findings indicate that the introduction of a preconcentration step during the measurements enables the detection of even trace-level changes in analyte concentration. The increased accumulation of the analyte at the electrode surface enhances the signal-to-noise ratio, facilitating clear differentiation of the target analyte from potential interfering species. This improvement not only enhances the reliability, accuracy, precision, and reproducibility of the measurement but also extends the applicability of the Cu-ZY/MWCNTs-GCE to real-world samples containing low AGO concentrations.

To date, only a limited number of studies have reported the use of voltammetric approaches for the electrochemical determination of agomelatine employing various modified electrodes (Table 3). In comparison, the Cu-ZY/MWCNTs-GCE demonstrates analytical performance that is not only comparable to but, in several key aspects, superior to previously reported sensors. Notably, the sensor exhibits exceptionally broad linear dynamic ranges, enabling accurate and reliable quantification of agomelatine from low nanomolar to submicromolar concentrations without the need for electrode reconditioning. This wide working range minimizes procedural complexity and enhances the reproducibility of measurements. Moreover, the Cu-ZY/MWCNTs-GCE demonstrates remarkable versatility, with successful application across multiple real matrices, including pharmaceutical tablets, urine, human plasma, and wastewater, underscoring its strong potential for both clinical and environmental monitoring of agomelatine.

Beyond its favorable analytical performance, the Cu-ZY/MWCNTs-GCE offers important practical advantages over previously reported sensing platforms. The modified electrode is fabricated through a simple, rapid, and cost-effective drop-casting procedure that does not require sophisticated equipment, expensive chemicals, or a multistep synthesis procedure. Importantly, the modified electrode is immediately ready for measurement after solvent evaporation, with no requirement for additional electrochemical activation. These features highlight the operational convenience, economic benefits, and robustness of the proposed Cu-ZY/MWCNTs-GCE, supporting its suitability for routine electrochemical determination of agomelatine across a diverse array of sample types.

### 3.4. Stability, Reproducibility and Selectivity of Cu-ZY/MWCNTs-GCE

The synergistic combination of the unique features of Cu-ZY and MWCNTs leads to the development of the AGO sensing platform, which exhibits excellent selectivity, stability, and reproducibility. The short-term stability and repeatability of Cu-ZY/MWCNTs-GCE were confirmed by relative standard deviation (*RSD*) values of 1.9% and 2.7% obtained for four consecutive voltammograms recorded in 0.04 mol L^−1^ BRB solution (pH 3.0) at AGO concentrations of 140 µg L^−1^ and 10 µg L^−1^, respectively. After storage under laboratory conditions for 7, 14, and 21 days, the peak current for both AGO concentrations changes by less than 16.5%, demonstrating the excellent long-term stability of the sensor. The reproducibility of the sensor was further evaluated using ten independently prepared electrodes (Section 2.3.2), for which the *RSD* of 9% for the analytical signal confirmed the consistent voltammetric response and reliability of the Cu-ZY/MWCNTs-GCE fabrication procedure.

Selectivity studies were carried out to evaluate the sensor’s ability to distinguish the target analyte from potentially interfering substances. The electrode response was tested in the presence of common coexisting species, including pharmaceutical excipients, typical biomolecules, and surface-active compounds. Consequently, DPV measurements were performed for 80 µg L^−1^ of AGO solution in the presence of a 10–2000-fold excess of inorganic ions (K^+^, Na^+^, Ca^2+^, Mg^2+^, Cl^−^, NO_3_^−^, SO_4_^2−^, PO_4_^3−^), a 5–125-fold excess of surfactants (SDS, CTAB, and Triton X-100), and a 10–250-fold excess of humic acid (HAS) and uric acid (UA). To assess the influence of common tablet excipients (titanium dioxide, starch, magnesium stearate, and cellulose), individually weighed portions of 2–6 mg were introduced directly into the voltammetric cell containing 80 µg L^−1^ of AGO. The substance tested was considered to exhibit an interference effect when its presence caused a change in the AGO peak current (*δ*) exceeding ±10% of its initial value, or when a noticeable distortion of the peak shape and/or a shift in peak potential was observed.

**Figure 5:**
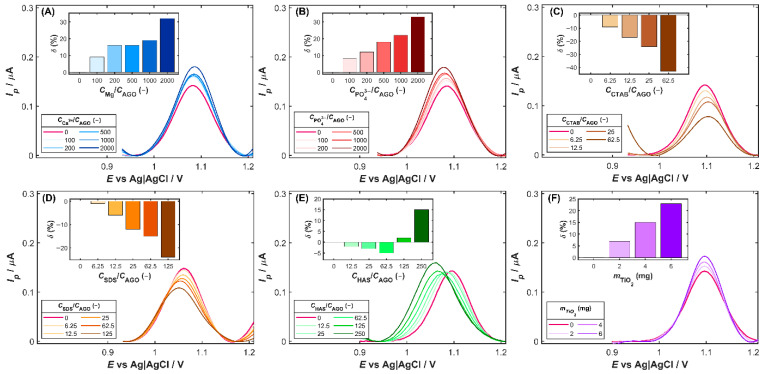
The influence of (**A**) magnesium ions, (**B**) phosphate ions, (**C**) cetrimonium bromide (CTAB), (**D**) sodium dodecyl sulfate (SDS), (**E**) humic acids (HAS), and (**F**) titanium dioxide on the DPV oxidation signal of AGO (80 µg L^−1^) at the Cu-ZY/MWCNTs-GCE. Insets: percentage change in peak current (*δ*) as a function of interferent excess. Supporting electrolyte: 0.04 mol L^−1^ BRB solution (pH 3.0). Experimental conditions as in Figure 4A.

Among inorganic ions, the most pronounced effect on the AGO oxidation signal is exhibited by magnesium and phosphate ions (Figure 5A and Figure 5B, respectively). Both ions produced a slight improvement in the anodic peak current with increasing interferent-to-analyte ratios. In the presence of Mg^2+^, the oxidation current gradually increased, reaching approximately 130% of the initial value at the highest excess of magnesium. A similar trend was observed for phosphate ions (Figure 5B), which caused an increase of up to 30% in the initial peak current. The observed current enhancement in the presence of Mg^2+^ or PO_4_^3−^ may be attributed to improved ionic conductivity and stabilization of the electrode/solution interface, possibly facilitating charge transfer during the oxidation process.

Of the surfactants examined, the cationic CTAB and the anionic SDS have distinct effects on the oxidation signal of agomelatine. The presence of CTAB (Figure 5C) led to a noticeable decrease in the anodic peak current of AGO with increasing surfactant concentration, reducing it to about 57% of the initial value at the highest CTAB level tested. A similar but less intense effect was observed for SDS (Figure 5D), which decreased the oxidation current to about 67% of the initial value. In both cases, suppression of the oxidation signal is probably associated with the adsorption of surfactant molecules onto the electrode surface, which form interfacial layers that hinder the electron transfer. In the case of CTAB, the positively charged headgroups may interact electrostatically with the electrode surface or the oxidized form of AGO, while the negatively charged SDS molecules can form a compact film that limits analyte access to the electroactive sites. The presence of humic acids (HAS), naturally occurring organic substances in aquatic and terrestrial environments, resulted in a gradual suppression of the AGO oxidation signal at excess levels ranging from 12.5 to 65.5 times the analyte concentration (Figure 5E). This behavior suggests the formation of a weakly conductive film of adsorbed humic macromolecules that partially hinders charge transfer and diffusion of AGO to the active side on the Cu-ZY/MWCNTs-GCE surface. However, beyond a 125-fold excess of HAS, a pronounced enhancement of the anodic signal occurred, which may be attributed to structural rearrangements and aggregation of humic acids at elevated concentrations, resulting in the pre-concentration of AGO through hydrophobic interactions with humic components. Furthermore, a shift in the AGO oxidation potential towards less positive values in the presence of humic acids was observed, due to weak interactions between AGO and humic macromolecules that facilitate its oxidation [34]. It should be noted that the influence of surface-active compounds and organic matter is expected to be negligible in typical environmental waters, where their concentrations are at nanomolar levels. Consequently, the voltammetric determination of agomelatine in most environmental matrices is largely unaffected by these substances.

In the case of the excipients commonly present in pharmaceutical formulations, starch, cellulose, and magnesium stearate caused negligible enhancement of the AGO oxidation signal, remaining within the acceptable tolerance range. On the contrary, titanium dioxide induced more pronounced changes in the AGO peak current (Figure 5F). At the highest amount of TiO_2_ tested (6 mg) introduced into the voltammetric cell, the DVP signal increased by approximately 25% relative to its initial value. This effect may arise from the adsorption of AGO molecules on the TiO_2_ surface and catalytic or capacitive interactions that enhance electron transfer at the electrode interface. Additionally, the increased surface heterogeneity caused by TiO_2_ particles likely facilitates charge transfer, thus strengthening the AGO oxidation signal [35].

To minimize the impact of matrix components on the voltammetric response of AGO, appropriate sample pretreatment and calibration strategies can be employed. Filtration or centrifugation effectively removes suspended solids and particulate matter, while solid-phase extraction (SPE) allows partial elimination of surfactants and humic macromolecules from the solution. Additionally, the standard addition method can be applied to compensate for residual matrix effects, ensuring reliable quantification under real-sample conditions.

### 3.5. Analysis of the Real Samples

**Figure 6:**
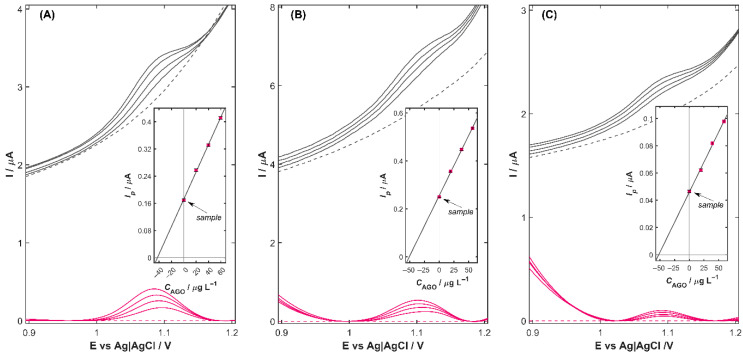
(**A**) Determination of AGO in *Agomelatine Adamed* tablets. (**B**) Study of the AGO recoveries in the CRM of *Waste Water SPS-WW2* and (**C**) in *human serum*. Inset: corresponding standard addition plot. Black lines—experimental curves, pink lines—voltammograms after background subtraction, dashed—DP curves recorded for blank.

An analysis of real samples using the developed protocol was carried out to assess its practical applicability. The performance of the Cu-ZY/MWCNTs-GCE in complex matrices (Figure 6) was evaluated by analyzing pharmaceutical tablets, as well as spiked samples of certified reference materials (CRM) of human serum, urine, and wastewater. To minimize matrix effects, the quantification was performed according to the standard addition method (Section 2.3.3), while the baseline correction step was realized by subtracting the background signal measured in the supporting electrolyte without the analyte (dashed line in Figure 6), resulting in colored-marked analytical curves. The accuracy of the proposed method was confirmed by calculating the relative error (*RE*) for pharmaceutical samples and the recovery values for spiked CRM wastewater and biological fluid samples (Table 4).

As shown in Figure 6, all tested samples exhibited well-defined AGO oxidation signals with no interference from matrix constituents, demonstrating the high selectivity and interference-resistant characteristics of Cu-ZY/MWCNTs-GCE. The results obtained for the commercial tablet showed good agreement with the labeled content, with an *RE* of 7.2% and an *RSD* of 3.0%, indicating that the excipients do not adversely affect the electrochemical response of the proposed sensor. For CRM analysis of wastewater, human serum and urine spiked with AGO, the calculated recoveries ranged from 97.8% to 108.8%, highlighting the robust performance and capacity of the Cu-ZY/MWCNTs-GCE for reliable and accurate detection of trace-level agomelatine within diverse and complex matrices. Overall, the successful quantification of agomelatine in real samples clearly demonstrates the robustness and practical relevance of the developed sensing platform. Its sensitivity, operational simplicity, and compatibility with pharmaceutical, clinical, and environmental samples position Cu-ZY/MWCNTs-GCE as a highly advantageous tool for routine monitoring, trace-level detection, and real-world analytical applications.

## 4. Conclusions

In this study, a novel Cu-ZY/MWCNTs-GCE was successfully developed and thoroughly characterized for the electrochemical determination of agomelatine in real samples. The integration of Cu-exchanged zeolite Y with multi-walled carbon nanotubes significantly improved electrode conductivity, catalytic activity, and electron transfer kinetics, resulting in markedly improved sensitivity and selectivity toward AGO. The proposed sensing platform demonstrated excellent analytical performance, including an exceptionally broad linear range, a detection limit at the nanomolar level, high selectivity against potential interfering species, and a rapid response time. The ability to preconcentrate AGO on the electrode surface further facilitated the detection of low-concentration samples, highlighting its suitability for trace-level analysis. Moreover, the electrode exhibited good reproducibility and long-term stability, confirming its robustness and practical applicability. Importantly, the fabrication procedure of the Cu-ZY/MWCNTs-GCE is straightforward, reproducible, and cost-effective, making it a viable tool for routine pharmaceutical and biological analysis. The successful application to pharmaceutical tablets, urine, human plasma, and wastewater analysis underscores the versatility of the sensor in diverse sample matrices. Overall, the developed Cu-ZY/MWCNTs-GCE represents a simple, highly efficient, and reliable platform for sensitive and selective electrochemical detection of AGO, with potential applicability to other pharmaceutical compounds in real-sample analysis.

## Figures and Tables

**Table 1:** Textural parameters of employed zeolite Y and MWCNTs.

Parameter	Unit	Material	
		Zeolite Y	MWCNTs
BET surface area	m^2^ g^−1^	1001.8	88.4
Micropore area	m^2^ g^−1^	992.6	15.9
External surface area	m^2^ g^−1^	9.2	72.5
Micropore and mesopore volume	cm^3^ g^−1^	0.40	0.37
Mesopore volume	cm^3^ g^−1^	0.37	0.01
Average pore diameter (BET)	nm	0.75	0.98
Average pore width (BJH)	nm	6.8	16.02

**Table 2:** Analytical parameters obtained for agomelatine determination using the DPV technique.

Parameter	Unit	*t_acc_* = 0 s	*t_acc_* = 60 s
Linear range *LR*	mol L^−1^ (µg L^−1^)	8.2 × 10^−8^–4.9 × 10^−7^ (20.0–118.6)	8.2 × 10^−9^–7.3 × 10^−8^ (2.0–17.7)
		5.7 × 10^−7^–9.6 × 10^−7^ (138.1–234.4)	
Slope *b*	µA L µg^−1^	0.0036 ± 0.0001	0.0095 ± 0.0003
		0.0019 ± 0.0001	
Intercept *a*	µA	0.011 ± 0.005	0.008 ± 0.003
		0.207 ± 0.011	
*r*	-	0.9991	0.9965
		0.9983	
*LOD*	mol L^−1^ (µg L^−1^)	1.8 × 10^−8^ (4.4)	4.3 × 10^−9^ (1.04)
*LOQ*	mol L^−1^ (µg L^−1^)	5.4 × 10^−8^ (13.2)	1.3 × 10^−8^ (3.2)
		Figure 4A	Figure 4B

**Table 3:** Comparison with earlier reported electrochemical sensors of agomelatine.

Electrode	Technique	*LR* /mol L^−1^	*LOD* /mol L^−1^	Samples	Ref.
^1^ CeO_2_/g-C_3_N_4_/GCE	SWV	4.1 × 10^−9^–8.2 × 10^−8^	3.95 × 10^−9^	tablets	[12]
^2^ MWCNTs/C/T/MCPE	DPV	1.0 × 10^−9^–0.5 × 10^−6^	5.26 × 10^−10^	tablets, urine	[13]
		1.0 × 10^−6^–5.0 × 10^−4^			
^3^ Co-NPs/SP/MCPE	SWV	3.5 × 10^−10^–1.0 × 10^−7^	2.12 × 10^−10^	human plasma	[14]
		2.0 × 10^−7^–1.0 × 10^−4^			
^4^ FeZn LDH/graphene/PANI/ GCE	SWV	5 × 10^−9^–140 × 10^−9^	9 × 10^−9^	tablets, urine	[15]
^5^ Cu@SNW_1_/GCE	SWASV	0.007× 10^−6^–1.00 × 10^−6^	2.0 × 10^−9^	urine, serum, saliva	[16]
		1.00 × 10^−6^–6.50 × 10^−6^			
^6^ Cu-ZY/MWCNTs-GCE	DPV	8.2 × 10^−9^–7.3 × 10^−8^	4.3 × 10^−9^	tablet, urine, human plasma, wastewater	This work
		8.2 × 10^−8^–4.9 × 10^−7^			
		5.7 × 10^−7^–9.6 × 10^−7^			

^1^ CeO_2_ nanoparticles/g-C_3_N_4_ sheets modified GCE; ^2^ multiwalled carbon nanotubes/cellulose/tween reformed carbon paste electrode; ^3^ cobalt nanoparticles/sugar polymer modified carbon paste electrode; ^4^ FeZn-layered double hydroxide/graphene/polyaniline modified GCE; ^5^ copper nanoparticles incorporated into a Schiff base network1 modified GCE; ^6^ Cu-zeolite Y/multi-walled carbon nanotubes modified GCE. SWV—Square-Wave Voltammetry; SWASV—Square-Wave Anodic Stripping Voltammetry.

**Table 4:** Results of agomelatine determination in real samples.

Sample	Amount of AGO/mg per Tablet		*RE*/%	*RSD*/%
	Declared	Found $\bar{x}$ ± *SD*		
*Agomelatine Adamed*	25	26.8 ± 0.8	7.2	3.0
**Sample**	**Concentration of AGO/µg L^−1^**		**Recovery/%**	***RSD*/%**
	**Added**	**Found**$\;\bar{x}$ ± ***SD***		
*Waste Water SPS-WW1*	50.0	54.4 ± 1.3	108.8	2.4
*Waste Water SPS-WW2*	50.0	53.5 ± 1.6	107.0	3.0
*Human serum*	50.0	52.4 ± 1.8	104.8	3.4
*Urine*	50.0	48.9 ± 2.3	97.8	4.7

$\bar{x}$—mean value (*n* = 3); *SD*—standard deviation; *RSD* = *SD*/$\bar{x}$·100%.

## Data Availability

The original contributions presented in this study are included in the article. Further inquiries can be directed to the corresponding author.
